# Sleep problems and parental stress among caregivers of children and adolescents enrolled in a digital mental health intervention

**DOI:** 10.3389/frcha.2023.1265095

**Published:** 2023-10-06

**Authors:** Landry Goodgame Huffman, Darian Lawrence-Sidebottom, Jennifer Huberty, Rachael Guerra, Monika Roots, Kurt Roots, Amit Parikh

**Affiliations:** ^1^ Bend Health, Inc., Madison, WI, United States; ^2^ FitMinded, Inc., Phoenix, AZ, United States

**Keywords:** telehealth, online coaching, online therapy, insomnia symptoms, pediatrics, family-based care

## Abstract

**Introduction:**

Caregivers of children with mental health problems such as anxiety, depression, and attention-deficit/hyperactivity disorder often experience heightened sleep problems, largely due to their children's disrupted sleep, and increased parental stress. Evidence suggests that mental and behavioral health care for children and adolescents has the potential to positively affect their caregivers; however, this has not been investigated in the context of pediatric digital mental health interventions (DMHIs). Therefore, the current study used caregivers' self-report measures to determine whether caregivers whose children are involved in a DMHI exhibit improvements in sleep problems and parental stress after initiation of their children's care.

**Methods:**

Caregivers with a child or adolescent participating in behavioral coaching and/or therapy with Bend Health Inc., a pediatric DMHI that involves both the child and caregiver in care (e.g., coaching and therapy), were included in the study (*n* = 662). Caregiver insomnia severity and parental stress were reported approximately every 30 days using the Insomnia Severity Index (ISI) and Parental Stress Scale (PSS). Changes in symptoms were assessed by comparing caregivers' symptom scores from baseline to first assessment after starting care.

**Results:**

Among caregivers with elevated insomnia severity (*n* = 88) and parental stress (*n* = 119) at baseline, 77% showed improvements in sleep and 73% showed improvements in parental stress after the initiation of their child's care, with significant decreases in score from baseline to post-care (ISI: *t* 72 = −4.83, *P* < .001, *d *= 0.61; PSS: *Z* = −4.98, *P* < .001, *d *= 0.59).

**Discussion:**

While extant research suggests ongoing links between child behavioral problems, parent sleep, and parent well-being, this is the first study to demonstrate improvements in caregiver sleep and stress when a child's mental health symptoms are addressed with behavioral care. Our findings offer promising preliminary evidence that caregivers experience significant secondary benefits to their sleep and parental stress when their children participate in a pediatric DMHI. Further research is warranted to investigate additional moderating and mediating factors, such as caregiver demographics and magnitude of child mental health improvement.

## 1. Introduction

There is mounting evidence that the relationship between the well-being of a child and their caregiver is complex and bi-directional, such that a child's well-being may be a salient predictor of their caregiver's well-being (1–8). Caregivers of children with mental health problems tend to also have impairments in their health and well-being (3, 9, 10). In fact, caring for a child with a mental health or behavioral problem has been identified as a risk factor for sleep problems and parental stress [i.e., stress related to the functioning of a caregiver (1, 11–13)].

Sleep is foundational to mental and behavioral health, supporting cognitive functioning, emotional regulation, development, and resilience in both children and adults (14–19). Studies have demonstrated that impairments in child sleep may predict caregiver sleep problems (5, 9, 20, 21). For example, caregivers lose sleep when their children awaken frequently at night, often because they need to help their child return to sleep or their sleep environment is disturbed by child-elicited factors such as noise and light (8). Sleep problems may be more prevalent for caregivers of children and adolescents with mental health problems, as these children tend to have more difficulty falling asleep, staying asleep, and waking up in the morning (22–24).

Research suggests that a child's mental health problems are also associated with parental stress. Children and adolescents with internalizing (e.g., anxiety and depression) and externalizing (e.g., conduct disorder and oppositional defiance) problems are more likely to have parents with elevated stress (1, 11, 13). Several studies have found that caring for a child with severe externalizing symptoms is particularly burdensome for caregivers and confers risk for increased parental stress (5, 6, 11, 13, 20, 25). Caregivers of children with mental health problems may experience increased strain on their time and finances, as well as diminished feelings of happiness and fulfillment in their parenting roles, both of which contribute to increased parental stress.

Interventions to address child sleep problems may improve caregiver sleep (26–28), even when caregiver sleep is not directly targeted by the treatment. Behavioral care (e.g., coaching and therapy) is a common treatment for mental health problems in children and adolescents, and there is evidence that the positive effects of in-person therapy for children with behavioral problems may also be associated with reductions in parental stress (29). Caregivers are often crucial to the initiation of their children's help-seeking and engagement in mental health care, and they often show strong feelings of relief as a result of their children's initiation in care (30, 31). Together, these studies suggest that interventions targeted to children and adolescents may confer secondary benefits to their caregivers.

Although traditional modalities of mental health care, such as in-person therapy, offer clear therapeutic benefits, many young people with mental health problems are not receiving the care they need due to provider shortages, geographic limitations, and associated stigma. In response to this lack of accessibility, digital mental health interventions (DMHIs) have grown more popular as a comparable modality for mental health care. Although the effectiveness of certain DMHI modalities has yet to be established (33), several meta-analyses have demonstrated that DMHIs, particularly those using computerized cognitive behavioral therapy and those with an in-person element, are a promising avenue to address anxiety and depression in children and adolescents (31–33). However, no studies to date have assessed whether a child's participation in care with a DMHI is also associated with positive outcomes for their caregiver. The purpose of this study is to assess whether caregivers with elevated sleep problems and parental stress show symptom improvements between their child's enrollment and first care appointment with a collaborative care digital mental health provider. We hypothesized that caregiver sleep problems and parental stress would decrease from baseline to the first assessment after beginning care and would continue to decrease throughout care.

## 2. Materials and methods

### 2.1. Design and participants

Caregivers (e.g., parents) of children (ages 2–12 years) and adolescents (ages 13–17 years) were eligible to be in the study if they: (1) began care with Bend Health Inc., a collaborative care DMHI, between January 1st 2023 and July 1st 2023, (2) had at least one coaching session with Bend Health Inc., and (3) completed caregiver assessments before and after the initiation of care. As such, *N* = 662 were eligible for inclusion in this study. Study procedures were approved by an independent institutional review board, Biomedical Research Alliance of New York (BRANY IRB; study identification number 23-12-034-1374).

### 2.2. Treatment

Bend Health, Inc. is a collaborative care DMHI that provides behavioral care for children and adolescents ages 2–17 years, an age range which encompasses peak ages of onset for most major mental illnesses (32). Bend Health also provides care to youths' families (e.g., parents), as described in detail previously (33, 34). Members can be referred to Bend Health Inc. via their PCP, and they can also enroll through insurance, employer benefits, or direct-to-consumer. Once enrolled in care, members and their caregivers are assigned a Behavioral Care Manager (BCM), who meets with the member and their caregiver in an initial synchronous evaluation (start of care). After the initial evaluation, the BCM continues to monitor and oversee the member's care with a Bend Health Inc. care team. All members are assigned a coach, and some are also assigned a therapist based on a member's symptom severity, mental health comorbidities, insurance coverage, and services desired. Members with more severe symptoms may have sessions with a therapist and a coach, whereas members with less severe symptoms may only have sessions with a coach. Members that require evaluation for psychiatric medication may also see a psychiatric provider (psychiatrist or nurse practitioner). Members may attend up to three synchronous (video and voice) sessions a month with a coach or therapist, and up to five synchronous sessions a month with any Bend Health Inc. practitioner (including psychiatric providers). Online asynchronous messaging is available for caregivers to communicate with their child's care team outside of synchronous sessions. Approximately once a month, caregivers and their child/adolescent are prompted to complete screeners and validated assessments of caregiver and child mental health outcomes (described in detail below).

In synchronous coaching and/or therapy sessions, Bend Health Inc. practitioners guide members and their caregiver/s through structured care plans that are designed to target specific symptoms and mental health problems (e.g., problematic behaviors and anxiety). The care programs are also delivered through an online learning resource center (including informational resources and guided activities), where members and their caregiver/s may further develop their skills to manage and improve mental health symptoms between synchronous sessions. Therapy sessions may also serve to provide a clinical foundation to the treatment of more serious or complex mental health challenges in children and adolescents. Bend Health Inc. therapists are licensed (LMFT, LPC, LCSW, or LMHC), coaches hold an ICF Coaching certification, NBC-HW, or master's degree in psychology, and BCMs hold a bachelor's degree in psychology or coaching certification. Moreover, all coaches and therapists are trained in modalities of cognitive behavioral therapy, behavioral activation, parent management training, mindfulness-based cognitive therapy, motivational interviewing, and mindfulness-based stress reduction. Bend Health Inc. care plans and coaching/therapy sessions are intended to be age-appropriate, and care programs for younger children place more of an emphasis on caregiver participation. Conversely, coaching/therapy sessions with adolescent members may be less involved for caregivers. Caregivers are required to attend all synchronous sessions with their child aged 2–12, and they must be in the same general location (e.g., in the same house) as their adolescent child aged 13–17. All members are free to withdraw from participation in Bend Health Inc. services and research studies at any time.

### 2.3. Measures

All measures are administered online using caregivers' self-report. When enrolling in care with Bend Health Inc., caregivers provide demographic information, including date of birth, sex at birth (male, female, or other) and gender (male, female, transgender, non-binary, or other), and race/ethnicity for the participating member (child or adolescent). From January 2023 to May 25, 2023, the race/ethnicity options were: “American Indian or Alaska Native”, “Asian”, “Black or African American”, “Hispanic or Latino”, “Native Hawaiian or other Pacific Islander”, “White”, and “Other”. Starting May 26, 2023, these options were updated to better align the measure with U.S. census standards by including the following categories: “White”, “Black or African American”, “American Indian or Alaska Native”, “Chinese”, “Vietnamese”, “Native Hawaiian”, “Filipino”, “Korean”, “Japanese”, “Chamorro”, “Other Asian”, “Other Pacific Islander”, “Some other race or multi-racial”, “Mexican, Mexican Am., Chicano”, “Puerto Rican”, “Cuban”, “Another Hispanic, Latino, or Spanish origin”. Before their first synchronous session with a BCM, caregivers complete assessments to assess their own sleep problems and parental stress (baseline). Caregiver assessments are repeated approximately every 30 days after enrollment to continually monitor symptom severity throughout the duration of care with Bend Health Inc.

To flag caregivers with sleep problems, caregivers are asked to respond to the following screener question: “During the past two (2) weeks, how much (or how often) have you had problems sleeping–that is, trouble falling asleep, staying asleep, or waking up too early?” Responses are on a 5-item Likert scale (0 = Not at all, 4 = Nearly every day). If the response to this screener is 2 or greater (several days or more frequently), caregivers are prompted to complete the insomnia severity index [ISI; (35, 36)]. If their response to this screener is less than 2, they do not complete the ISI. The ISI is a validated assessment consisting of 7 items, in which three items query the severity of a specific sleep problem (e.g., difficulty falling asleep) and four items query satisfaction or perception of sleep difficulties (34). In response to each item, the caregiver selects the best-fit response on a 5-item Likert scale (e.g., 0 = None, 5 = Very severe) to indicate their sleep problems over the last two weeks.

For parental stress, caregivers respond to the following screener questions, taken from the Parental Stress Scale (PSS; 37): “The major source of stress in my life is my child” and “Having a child leaves little time and flexibility in my life.” Responses to the PSS items are on a 5-item Likert scale (1 = Strongly disagree, 5 = Strongly agree). If the response to either screener question is 3 (undecided) or greater, the caregiver is prompted to complete the other 16 items on the Parental Stress Scale. The PSS includes 18 items which are presented as statements of subjective feelings and perceptions of a caregiver's feelings about their relationship with their child and their role as a parent. Most items are framed in terms of negative experience [e.g., “The major source of stress in my life is my child(ren)”] and some are framed in terms of positive experience (e.g., “I am happy in my role as a parent”). Items 1, 2, 5, 6, 7, 8, 17, and 18 are frames as a positive experience.

### 2.4. Statistical analysis

Months in care was calculated as the number of months (30 days) since the start of care (first synchronous session). The number of care sessions per month was calculated as the total number of coaching and therapy sessions attended divided by the number of months in care. One month was used as the divisor for care sessions per month for members in care less than one month, to accurately represent care session frequency. Given that the race/ethnicity question was change part-way through the study timeframe, the following categories were used to report member's race/ethnicity information: White (includes response: “White”), Black or African American (includes response: “Black/African American”), Hispanic or Latino (includes responses: “Hispanic/Latino”, “Puerto Rican”, “Other Hispanic/Latino”, and “Mexican, Mexican American, or Chicano”), Asian (includes responses: “Asian”, “Other Asian”, “Chinese”, “Korean”, and “Filipino”), and Other or multi-racial (includes responses: “Other” and multi-select responses).

Caregiver ISI scores were calculated by aggregating the scores for all seven items, for an ISI score range of 0–28. An ISI score of 0–7 indicates no clinically significant insomnia, a score of 8–14 indicates subthreshold insomnia, a score of 15–21 indicates moderate severity clinical insomnia and a score of 22 or greater indicates severe clinical insomnia. To calculate caregiver PSS scores, the individual scores from the responses to the positively framed items (e.g., “I am happy in my role as a parent”) were reversed (e.g., a response of “1 = Strongly disagree” was converted to a score of 5). Then, all item scores were aggregated for a PSS score range of 18–90. Using the same scoring categories as described by others (36, 37), A PSS score of 18–41 indicates mild severity parental stress, a PSS score of 42–65 indicates moderate severity parental stress, and a score greater than 65 indicates severe parental stress.

#### 2.4.1. Member characteristics

Caregivers with an ISI score indicating clinically significant insomnia severity (moderately severe or greater; score > 14) at baseline were included in the “caregivers with elevated sleep problems” group. Given that there is no established criteria for clinically significant parental stress, caregivers with a PSS score indicating moderate or severe parental stress (score > 41) were included in the “caregivers with elevated parental stress” group. Only caregivers with an assessment before the start of care and after the start of care were included in all statistical analyses. The following between-groups tests were performed to compare member characteristics between each elevated symptom group and the non-elevated symptom group: Wilcoxon signed-rank tests for member age at baseline, duration in care, and session frequency between the non-elevated symptom group and each elevated symptom group, and Chi-squared tests for distributions of sex (female vs. non-female), gender conformity, race/ethnicity (White vs. non-white), mental health condition diagnosis (for no diagnosis, anxiety disorders, ADHD, and depressive disorders), and care type (coaching vs. coaching and therapy).

#### 2.4.2. Change in symptom severity

Only data from caregivers with elevated sleep problems or caregivers with elevated parental stress were included in analyses of symptom change. To assess whether caregiver sleep problems and parental stress changed from before to after the initiation of care with the DMHI, ISI and PSS scores from the assessment before the start of care were compared to scores from the first assessment after the start of care (post care) using *t*-tests or Wilcox signed-rank tests, as appropriate. Effect sizes for these statistical tests were reported using Cohen's *d* (38). To determine whether duration in care had a significant effect on sleep problems and parental stress, ISI and PSS scores over all months in care were assessed using linear mixed-effects models with a fixed effect of months in care and a random effect of subject on the intercept. Potential predictor variables were added to this main model individually in alternative models, and then each alternative model was tested against the main model using the likelihood-ratio test (LRT). Where a predictor improved model fit (significant LRT), it was retained in the final model. The following predictor variables were assessed: member age (at caregiver assessment), member sex (female vs. non-female), member race/ethnicity (white vs. non-white), and member care type (coaching vs. coaching & therapy). Results from the *F*-tests for each main effect are reported to assess for changes in symptom severity over months in care, and also to assess associations between retained predictor variables and symptom severity. Missing data was estimated using maximum likelihood (ML) estimation in the linear mixed-effects models.

All reported *P*-values were corrected for using the Benjamini-Hochberg method (39). The demographic and care statistics (between-groups comparisons) for each group of elevated symptoms (elevated sleep problems and elevated parental stress) were corrected as a group. Change statistics (pre-care vs. post-care) and all *P-*values from the linear mixed-effects model *F*-tests for each symptom type (sleep problems and parental stress) were corrected as a group. Percentages, mean and standard deviation (M ± SD), and median and interquartile range (IQR) were used to describe the data throughout, and the alpha level was set to 0.05 for all analyses.

## 3. Results

### 3.1. Baseline symptom severity

At baseline, over half of the caregivers screened out of completing the ISI assessment (52.6%; *n* = 348), 10.6% had no clinically significant insomnia (*n* = 70), 23.6% had sub-threshold (mild) insomnia (*n* = 156), and 13.3% had clinically significant insomnia (*n* = 88). Of those with clinically significant (elevated) insomnia, 75.0% had moderate severity insomnia (*n* = 66) and 25.0% had severe insomnia (*n* = 22). All caregivers that completed the ISI had a score of 11.97 ± 5.19 at baseline (*n* = 314). In terms of parental stress at baseline, 36.9% of caregivers screened out of completing the PSS assessment (*n* = 244), 45.2% had mild parental stress (*n* = 299), and 18.0% had moderate parental stress (*n* = 119). No caregivers had severe parental stress. Caregivers that completed the PSS had scores of 37.02 ± 8.16 at baseline (*n* = 498). Ultimately, 57 caregivers were only in the elevated sleep problems group, 88 were only in the elevated parental stress group, and 31 were in both groups. All other caregivers (*n* = 486) were in the non-elevated symptoms group.

### 3.2. Member characteristics

Comprehensive demographic characteristics and results from between-groups statistical tests are reported in Table 1. The elevated sleep problems group (13.3%; *n* = 88) was largely similar to caregivers with non-elevated symptoms (non-elevated sleep problems and parental stress; *n* = 486) in terms of their child or adolescent member's age, gender conformity, and ethnicity (all *P* > .05). Caregivers with sleep problems had an approximately equal split of female versus non-female members (Female: 47.7%; *n* = 42). Most caregivers with elevated sleep problems reported their member's race/ethnicity as “White” or other/multi-racial (together 82.9%; *n* = 73). In terms of mental health conditions of children/adolescents of caregivers with elevated sleep problems, 27.3% (*n* = 24) had an anxiety disorder, 28.4% (*n* = 25) had a subtype of ADHD, and 4.5% (*n* = 4) had a depressive disorder. Compared to the non-elevated symptoms group, the rates of diagnosis were similar for children of caregivers in the elevated sleep problems group.

**Table 1:** Member characteristics and results from statistical comparisons between each group of caregivers with elevated symptoms and caregivers with non-elevated symptoms.

Member characteristics	Non-elevated symptoms group (*n* = 486)	Elevated sleep problems group (13.3%; *n* = 88)			Elevated parental stress group (18.0%; *n* = 119)		
	Value	Value	Statistic	*P*-value	Value	Statistic	*P*-value
Age in years	10.9 ± 3.7	10.0 ± 3.5	−2.20^a^	.23	10.1 ± 3.4	−2.55^a^	*0*.*061*
Sex							
Female	53.1% (*n* = 258)	47.7% (*n* = 42)	0.66^b^	.72	40.3% (*n* = 48)	5.72^b^	*0*.*062*
Male	45.9% (*n* = 223)	51.1% (*n* = 45)			58.8% (*n* = 70)		* *
Other or non-binary	1.0% (*n* = 5)	1.1%% (*n* = 1)			0.8% (*n* = 1)		* *
Gender conformity							
Conforming	93.2% (*n* = 453)	94.3% (*n* = 83)	0.02^b^	.88	94.1% (*n* = 112)	0.02^b^	0.95
Non-conforming	6.8% (*n* = 33)	5.7% (*n* = 5)			5.9% (*n* = 7)		
Race/ethnicity							
White	40.9% (*n* = 199)	38.6% (*n* = 34)	0.08^b^	.88	42.9% (*n* = 51)	0.08^b^	0.95
Other or multi-racial	45.5% (*n* = 221)	44.3% (*n* = 39)			42.0% (*n* = 50)		
Black/African American	5.8% (*n* = 28)	6.8% (*n* = 6)			5.0% (*n* = 6)		
Hispanic/Latino	4.9% (*n* = 24)	2.3% (*n* = 2)			2.5% (*n* = 3)		
Asian	2.9% (*n* = 14)	8.0% (*n* = 7)			7.6% (*n* = 9)		
Mental health condition							
None	17.9% (*n* = 87)	23.9% (*n* = 21)	1.37^b^	.58	22.7% (*n* = 27)	1.14^b^	0.54
Anxiety disorder	41.4% (*n* = 201)	27.3% (*n* = 24)	5.63^b^	.**58**	25.2% (*n* = 30)	9.89^b^	**0.022**
ADHD	18.3% (*n* = 89)	28.4% (*n* = 25)	4.16^b^	.**23**	21.0% (*n* = 25)	0.30^b^	0.81
Depressive disorder	7.4% (*n* = 36)	4.5% (*n* = 4)	0.55^b^	.72	4.2% (*n* = 5)	1.09^b^	0.53
Care type							
Coaching only	80.9% (*n* = 393)	75.0% (*n* = 66)	1.25^b^	.58	75.6% (*n* = 90)	1.32^b^	0.54
Coaching and therapy	19.1% (*n* = 93)	25.0% (*n* = 22)			24.4% (*n* = 29)		
Care statistics							
Months in care	2.3 ± 1.2	2.4 ± 1.3	−0.31^a^	.88	2.4 ± 1.2	−0.77^a^	0.70
Care sessions per month	1.6 ± 0.8	1.6 ± 0.9	−0.26^a^	.88	1.6 ± 0.7	−0.06^a^	0.95

The between-groups comparison for sex was performed using the categories female and non-female. The between-groups comparison for race/ethnicity was performed using the categories white and non-white. *P* values have been corrected for multiple comparisons using the Benjamini-Hochberg method. Statistically significant values are bolded (*P* < .05), and statistical trends are italicized (*P* < .10).

^a^ Z statistic.

^b^ χ^2^ statistic.

Compared to caregivers with non-elevated symptoms, the elevated parental stress group (18.0%; *n* = 119) tended to have younger children (*Z* = −2.55, *P* = .061) and their child/adolescent members were less predominantly female (*χ*^2^ = 5.72, *P* = .062), though the between-groups statistical tests approached significance. These two groups did not differ in terms of gender conformity and race/ethnicity. Caregivers with elevated parental stress more predominantly had children vs. adolescents (73.9%; *n* = 88), less than half had female children (41.1%; *n* = 48), and most reported their member's race/ethnicity as “White” or other/multi-racial (together 84.9%; *n* = 101). In terms of mental health conditions of children/adolescents of caregivers with elevated parental stress, 25.2% (*n* = 27) had an anxiety disorder, 21.0% (*n* = 25) had a subtype of ADHD, and 4.2% (*n* = 5) had a depressive disorder. Compared to caregivers with non-elevated symptoms, caregivers with elevated parental stress were less likely to have children with an anxiety disorder diagnosis (*χ*^2 ^= 9.89, *P* = .022). Otherwise these two groups did not differ in terms of depressive disorder, ADHD, or no diagnosis.

For all caregivers, most of their children/adolescents were in coaching only vs. coaching and therapy: 80.9% for caregivers with non-elevated symptoms (*n* = 393), 75.0% for caregivers with elevated sleep problems (*n* = 66), and 75.6% for caregivers with elevated parental stress (*n* = 90). Children and adolescents of caregivers with non-elevated symptoms were in care for 2.32 ± 1.17 months (range: 0.20–5.43 months) and members had 1.58 ± 0.78 care sessions (coaching and therapy) per month. This duration in care did not differ between caregivers with non-elevated symptoms and caregivers with elevated sleep problems (2.36 ± 1.27 months), as well as caregivers with elevated parental stress (2.40 ± 1.24 months). The number of care sessions per month also did not differ across groups (Elevated sleep problems: 1.57 ± 0.92; Elevated parental stress: 1.56 ± 0.74). Caregivers completed between two and six assessments total during their child's care with the DMHI; Table 2. While in care with the DMHI, caregivers with elevated sleep problems completed the sleep assessment every 1.27 ± 0.38 months and caregivers with elevated parental stress completed the parental stress assessment every 1.25 ± 0.28 months.

**Table 2:** Number of assessments completed by caregivers with elevated sleep problems and caregivers with elevated parental stress.

Number of assessments completed	Elevated sleep problems group	Elevated parental stress group
	(*n* = 88)	(*n* = 119)
2	46.6% (*n* = 41)	46.2% (*n* = 55)
3	31.8% (*n* = 8)	25.2% (*n* = 30)
4	13.6% (*n* = 12)	21.8% (*n* = 26)
5	7.9% (*n* = 7)	6.7% (*n* = 8)

### 3.3. Change in symptom severity

#### 3.3.1. Sleep problems

**Figure 1:**
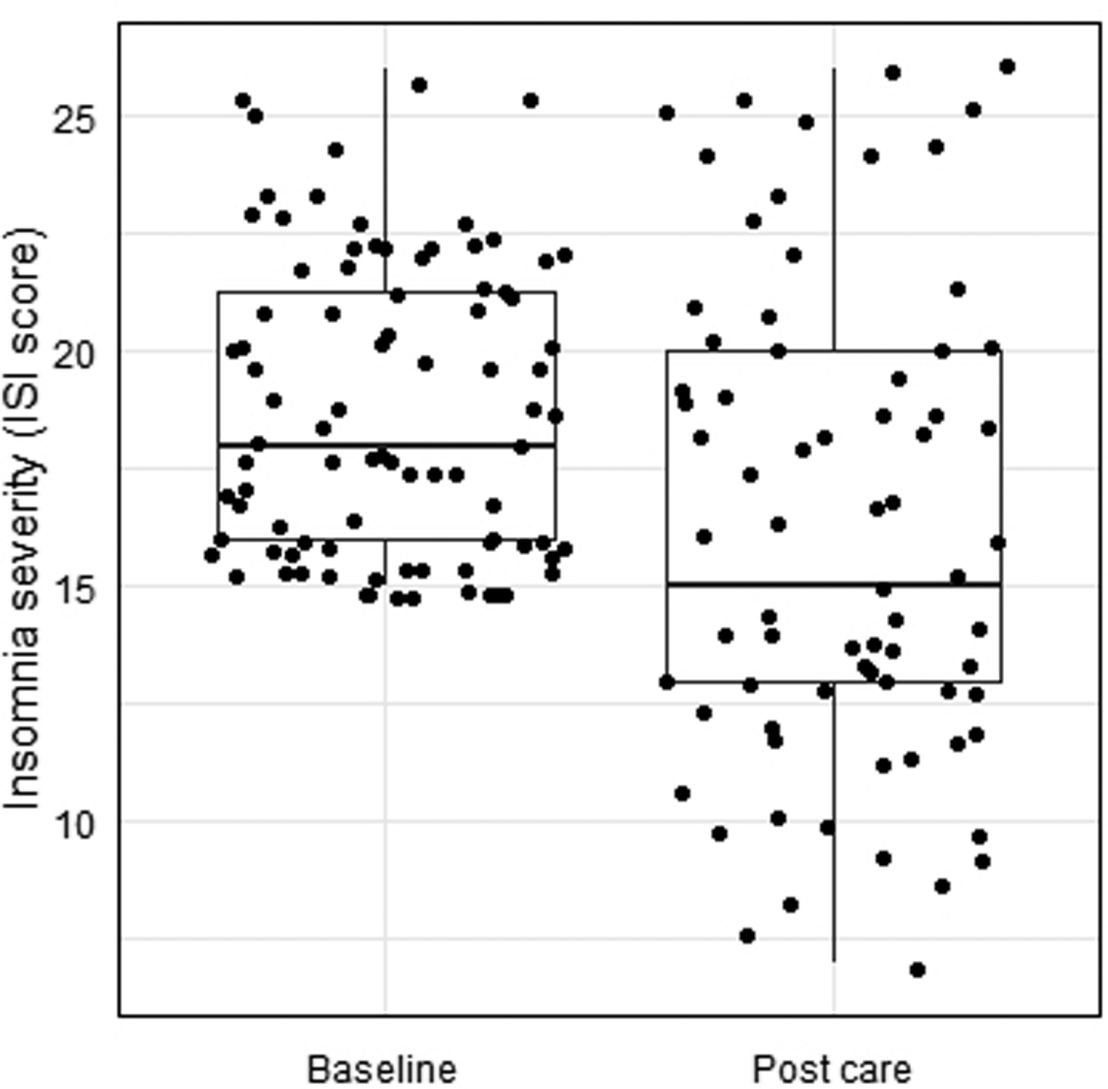
Caregiver insomnia symptom severity before and after the start of care with the DMHI.

Caregivers with elevated sleep problems had an ISI score of 18.75 ± 3.11 at baseline. On the first assessment after the start of care, 18.2% of caregivers with elevated sleep problems screened out of completing the ISI (*n* = 16), 38.6% had subthreshold insomnia symptoms (mild; *n* = 34), 29.5% had moderately severe insomnia symptoms (n = 26), and 13.6% had severe insomnia symptoms (n = 12). For caregivers with elevated sleep problems, 77.3% exhibited a decrease in insomnia severity from before to after the start of care (n = 68), as indicated by screening out of the first assessment after care start or by a decrease in ISI score from baseline. For those that completed the ISI on the first assessment after the start of care (*n* = 72), their ISI scores decreased from 18.81 ± 3.11 at baseline to 16.29 ± 4.96 after the start of care (*d = *0.61), for a change score of −2.59 ± 4.58 points from baseline (*t* 72 = −4.83, *P* < .001; CI: −3.66, −1.52); see Figure 1.

**Figure 2:**
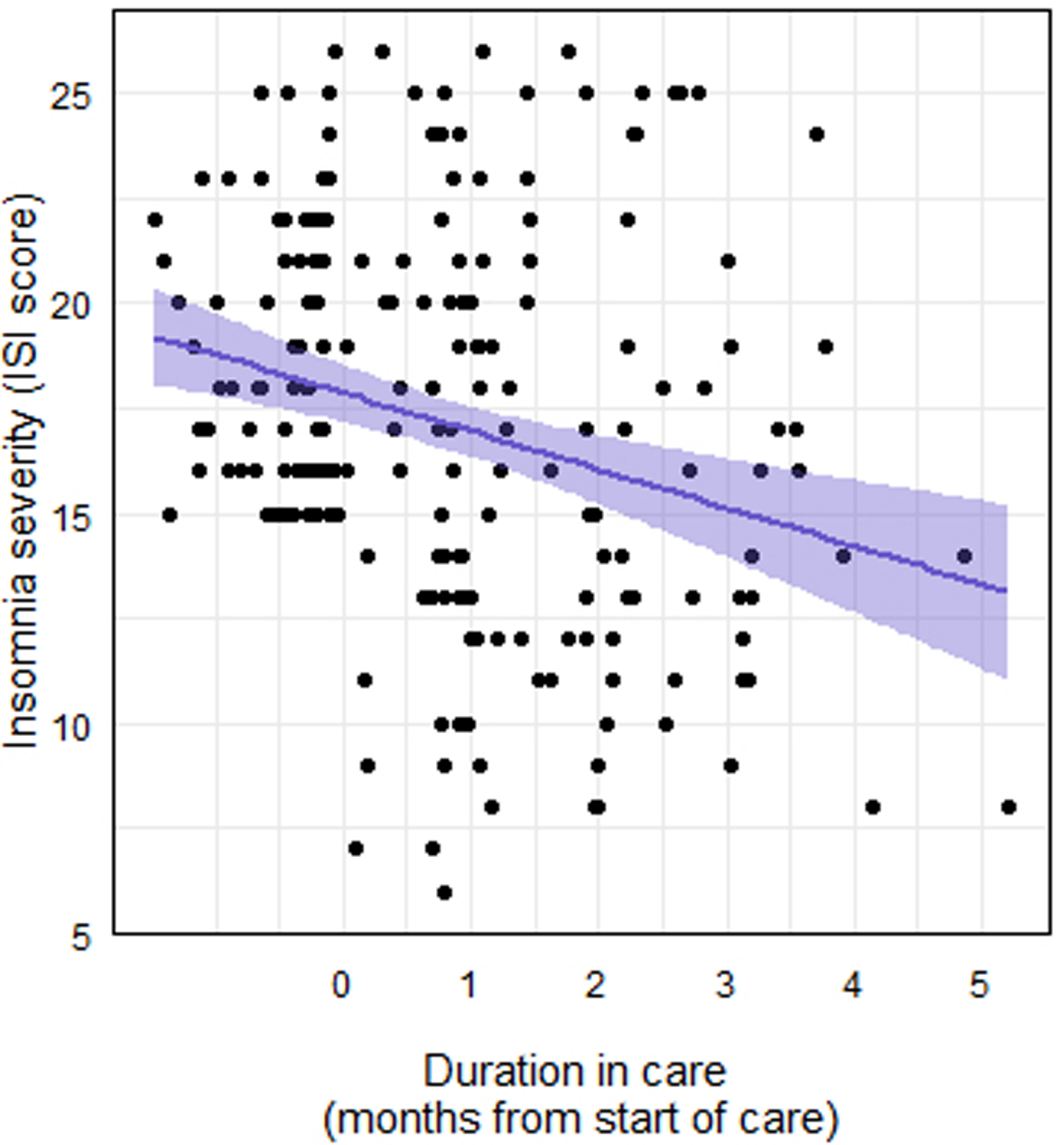
Caregiver ISI score over the course of their child's care with the DMHI.

The addition of the following child/adolescent characteristics as predictors did not improve the fit of the sleep model: age (*χ*^2^ = 2.29, *P* = .13), sex (female or non-female: *χ*^2^ = 1.46, *P* = .23), race/ethnicity (white or non-white: *χ*^2^ = 2.37, *P* = .12), and care type (coaching or coaching and therapy: *χ*^2^ = 1.79, *P* = .18). Thus, the final model included a fixed effect of months in care and a random effect of member ID on the intercept. Ultimately, caregiver ISI scores decreased significantly by months in care (*F*1,131 = 26.02, *P* < .001), such that each additional month in care was associated with a 0.95 point decrease in ISI score (Figure 2).

#### 3.3.2. Parental stress

**Figure 3:**
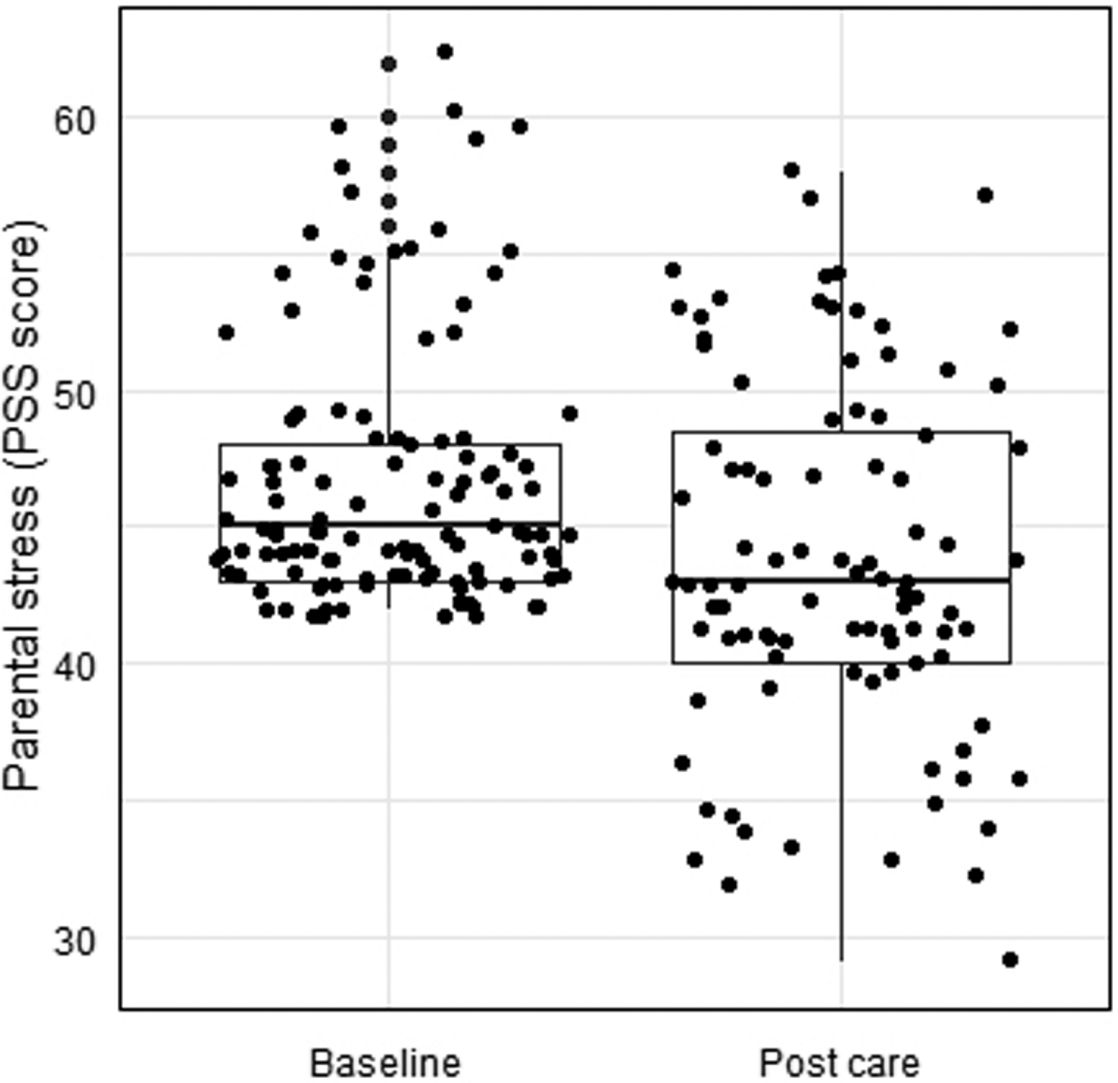
Caregiver parental stress before and after the start of care with the DMHI.

Caregivers with elevated parental stress had a PSS score of 46.74 ± 4.87 at baseline. On the first assessment after the start of care, 20.2% of caregivers previously indicated as having elevated parental stress screened out of completing the PSS (*n* = 24), 31.9% had mild parental stress (*n* = 38), and 47.9% had moderate parental stress (*n* = 57). For caregivers with elevated parental stress, 73.1% exhibited a decrease in parental stress from before to after the start of care (*n* = 87), as indicated by screening out of the first assessment after care start or by a decrease in PSS score from baseline. For those that completed the PSS on the first assessment after the start of care (*n* = 95), their PSS scores decreased from 47.24 ± 5.17 at baseline to 43.77 ± 6.49 after the start of care (*d *= 0.59; CI: −*Inf*, −2.50), for a median change score of −3 points (IQR = 7.25; *Z* = −4.98, *P* < .001); see Figure 3.

**Figure 4:**
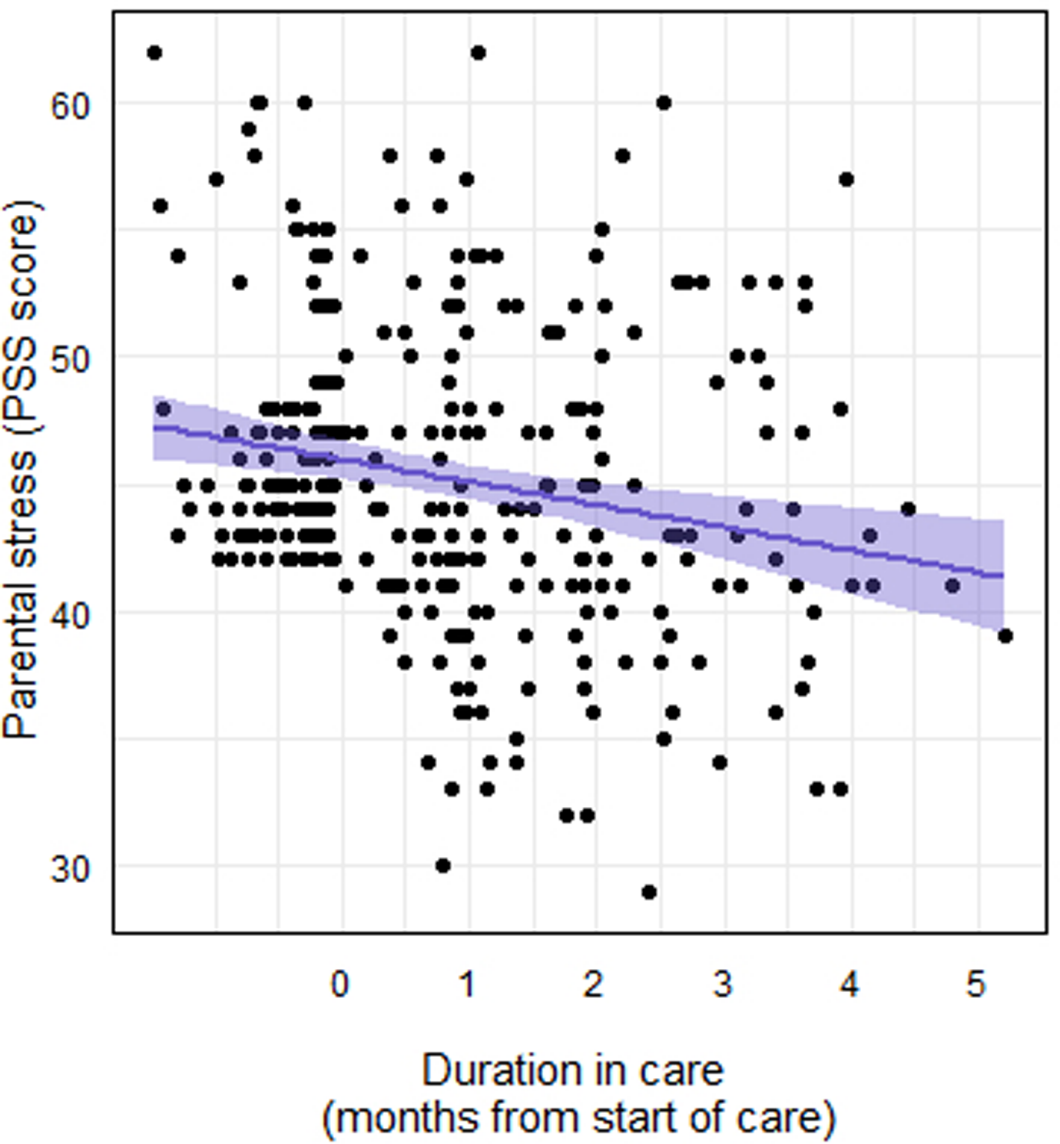
Caregiver PSS score over the course of their child's care with the DMHI.

The addition of the following child/adolescent characteristics as predictors improved the fit of the stress model: sex (female or non-female; *χ^2^* = 4.99, *P* = .025) and care type (coaching or coaching and therapy; *χ*^2^ = 9.03, *P* = .003). The addition of the following child/adolescent characteristics as predictors did not improve the fit of the stress model: age (*χ*^2^ = 2.08, *P* = .15) and race/ethnicity (white or non-white: *χ*^2^ = 2.30, *P* = .069). Thus, the final model included fixed effects of months in care, sex, and care type, and a random effect of member ID on the intercept. Caregiver PSS scores decreased significantly by months in care (*F*1,188 = 35.17, *P* < .001), such that each additional month in care was associated with a 1.14 point decrease in PSS score (Figure 4). The main effect of female sex approached statistical significance (*F*1,116 = 3.53, *P* = .063), as caregivers of females tended to have higher PSS scores than caregivers of non-females. The main effect of care type was statistically significant (*F*1,116 = 6.86, *P* = .013), such that caregivers of children in coaching only tended to have lower PSS scores than caregivers of children in coaching and therapy.

## 4. Discussion

### 4.1. Principal results

The purpose of this study was to assess caregivers' self-reported sleep quality and parental stress before the start of care and up to 6 months after the start of care for their child or adolescent with a collaborative care digital mental health provider, Bend Health, Inc. We found that sleep problems and parental stress of caregivers decreased after their child or adolescent initiated care in a collaborative care DMHI. Improvements in caregivers’ sleep problems and parental stress were linked to the duration of their children's participation with the DMHI, with each additional month in care associated with larger improvements. Although nascent research has demonstrated benefits to caregivers of youth participating in behavioral care (29, 40, 41), this is the first study to suggest that these benefits extend to digitally-delivered behavioral care.

Both sleep problems and parental stress decreased significantly between the child's initiation of care and first follow-up assessment among parents whose children were enrolled in Bend Health. Despite the fact that current Bend Health care programs are directed toward children and not parents, our findings suggest that initiation and duration of mental health care for children is positively linked to improvements in crucial aspects of caregiver well-being. These findings are bolstered by previous studies, which have found that parent sleep problems decrease as a result of behavioral care that mitigates child sleep problems (26, 27). Findings from several studies among parents of children with oppositional symptoms also suggest that parental stress decreases as a result of children's participation in behavioral intervention that mitigates their problem behaviors (29, 40, 41). While another recent study (30) also suggests ongoing links between child behavioral problems, parent sleep, and parent affect, this is the first to demonstrate improvements in caregiver sleep and stress when a child's mental health symptoms are addressed with behavioral care (e.g., coaching and/or therapy). Given that a caregiver's sleep and stress are closely related to their child's sleep (5, 9, 20, 21), and children with mental health problems tend to have difficulty sleeping (22–24), these findings may reflect the downstream effects of child mental health treatment and related symptom changes on their caregiver's well-being (8). In other words, parents' sleep and stress may be linked to the improvements their child exhibits throughout involvement in a DMHI. However, it should be noted that the link between parent and child well-being is complex and multi-faceted, and many other factors that may moderate this association were not considered, such as caregivers' coping strategies and social support (42, 43). As such, further research is necessary to determine the causal mechanisms underlying these associations.

The current study also suggests that sleep problems are more common among caregivers who enroll their children in a DMHI than those in the general population. While an estimated 14.5% of adults in the general population report trouble falling asleep most days or every day (44), 48% of caregivers in the current study reported at least some insomnia symptoms (trouble falling asleep, staying asleep, or waking up too early) and 14% had clinically significant insomnia symptoms. It may be that caregivers who are seeking care for their children are experiencing increased stress, which in turn contributes to their sleep problems. Although further research is necessary, these elevated rates nonetheless highlight the persistent link between child mental health and caregiver sleep problems. As DMHIs for children and adolescents continue to develop, our findings highlight not only the potential of but also the need for secondary benefits to caregivers' sleep and stress.

### 4.2 Limitations and future directions

Although promising, findings from the current study are limited by several factors. Firstly, our sample was limited to those participating in a collaborative care DMHI, and thus our findings may not generalize to caregivers whose children are engaged in a different type of DMHI or a more traditional modality for mental health treatment. Additionally, our assessment of caregiver sleep was limited to the subjective measure of the ISI. While objective measures of sleep such as wearable devices and polysomnography are more accurate measures of sleep quality, sleep-disordered breathing, and sleep timing, the ISI has been validated in various clinical and non-clinical populations against objective measures of sleep (45, 46). However, future research should include an objective measure of sleep to more holistically quantify sleep quality and timing of caregivers.

In our assessment of caregiver sleep problems, caregivers may screen out of completing the full validated ISI assessment based on their responses to the screening questions. As such, the current analyses did not include follow-up assessment data for members who exhibited low insomnia symptoms after their first assessment. This lack of data among those arguably exhibiting the largest improvements likely skewed our longitudinal analyses to primarily reflect those with more persistent and severe insomnia symptoms. To mitigate this potential selection effect, caregivers’ reductions in ISI scores or screening out on follow-up assessments were considered improvements in sleep problems. Moreover, our use of self-report only to report caregivers' sleep problems and parental stress may have not offered a complete picture of their symptoms. Further study is warranted using more objective measures of sleep (e.g., via sleep trackers) as well as child- or clinician-reported measures of parental stress.

Moreover, the current study did not include considerations of child symptom improvement throughout involvement at Bend Health, Inc. Future studies should assess whether improvements in caregiver sleep and parental stress may be predicted by changes in their child's symptom severity while receiving care from a DMHI. Previous studies have shown that caregiver sleep problems and parental stress are associated with their child's mental health problems (8, 25). Thus, better mental health outcomes of children in care with a DMHI may relate to larger improvements in their caregiver's sleep and parental stress. Alternatively, caregiver sleep and parental stress may improve regardless of their child's outcomes related to caregivers' feelings of relief and reduced stress when their children are receiving care. Assessing potential mechanisms underlying the positive association between child mental health and caregiver well-being, such as improvements in the parent-child relationship and reductions in family conflict.

Many potential covariates and confounding factors were not included in the present study. For example, we did not collect data on caregiver demographics such as sex, age, race and ethnicity, socioeconomic status, relationship to the child, marital status, and employment status. Caregiver involvement in parenting tends to vary based on caregivers' gender (with mothers typically more involved), socioeconomic status (parents of higher SES exhibit more involvement and warmth), and culture of origin (47–49). More specifically, caregiver participation and engagement in their children's mental health treatment specifically is positively linked to female gender, younger age, higher income, more education, and presence of both caregivers (43). We also did not consider other stressors that may impact caregivers' sleep and parental stress, such as psychiatric comorbidities, co-parenting, and work-related stressors (50). As such, further research is warranted.

Given the nascency of these research questions–especially as they pertain to DMHIs–it is unknown whether caregivers whose children are involved in DMHIs exhibit improvements in other indices of well-being, such as mental health symptoms, burnout, and productivity. These are crucial issues, given that an increasing number of caregivers are reporting poor mental health (51), feelings of burnout (52), and low workplace productivity due to their children's mental health (53, 54). In addition to these indices, future research should consider the associations between caregivers' well-being and their parenting behaviors among families involved in DMHIs. Caregivers with low levels of parental stress tend to utilize positive parenting techniques more regularly (4, 12), and these techniques in turn can be crucial in facilitating children's involvement in mental health treatment (55). Finally, randomized controlled trials comparing DMHI participants to non-DMHI therapy groups and waitlist controls may help establish the causal mechanisms between children's mental health treatment via DMHIs and caregivers' well-being.

### 4.3. Concluding remarks

The current study offers promising preliminary evidence that caregivers of youth engaged in a DMHI show marked improvements in their sleep problems and parental stress, and the magnitude of these improvements is positively associated with the duration of care. These findings suggest that among families involved in DMHIs, the mental health of children is closely tied to the well-being of their caregivers. They highlight a unique opportunity for DHMIs such as Bend Health, Inc. to maximize their effectiveness and scope by considering caregivers as not only sources of support for their children, but as potential beneficiaries of the intervention as well. Considering the close association between caregiver well-being and child mental health care, employers should also consider making health benefits more holistic by offering mental and behavioral health care for employees' children. Ultimately, these findings highlight the potential benefits of family-centered digital mental health care, in which caregivers' therapeutic needs are considered alongside the needs of their children.

## Data Availability

The data analyzed in this study is subject to the following licenses/restrictions: The data sets analyzed during the current study are not publicly available, as this would violate Bend Health Inc.'s privacy policy. However, aggregated and anonymized data that is not associated with individual users and does not include personal information is available from the corresponding author on reasonable request. Requests to access these datasets should be directed to darian.lawrence@bendhealth.com.
